# A novel concept of screening for subgrouping factors for the association between socioeconomic status and respiratory allergies

**DOI:** 10.1038/s41370-021-00365-x

**Published:** 2021-07-12

**Authors:** Christoph Muysers, Fabrizio Messina, Thomas Keil, Stephanie Roll

**Affiliations:** 1 Institute of Social Medicine, Epidemiology and Health Economics, Charité—Universitätsmedizin Berlin, corporate member of Freie Universität Berlin and Humboldt-Universität zu Berlin, Berlin, Germany; 2 Statistics and Data Insights, Bayer AG, Berlin, Germany; 3 Data Science, Bayer Pharmaceutical PLC, Reading, UK; 4 Institute for Clinical Epidemiology and Biometry, Julius-Maximillians-University Würzburg, Würzburg, Germany; 5 State Institute for Health, Bavarian Health and Food Safety Authority, Bad Kissingen, Germany

**Keywords:** “Subscreen” R package, Epidemiology survey, Asthma, Rhinitis

## Abstract

**Background:**

The new subgroup screening tool “subscreen” aims to understand the unclear and complex association between socioeconomic status (SES) and childhood allergy. This software R package has been successfully used in clinical trials but not in large population-based studies.

**Objective:**

To screen and identify subgrouping factors explaining their impact on the association between SES and respiratory allergies in childhood and youth.

**Methods:**

Using the national German childhood and youth survey dataset (KiGGS Wave 2), we included 56 suspected subgrouping factors to investigate the association between SES (low vs. high) and allergic rhinitis and/or asthma in an exploratory manner. The package enabled a comprehensive overview of odds ratios when considering the SES impact per subgroup and analogously all disease proportions per subgroup.

**Result:**

Among the 56 candidate factors, striking subgrouping factors were identified; e.g., if mothers were younger and in the low SES group, their children had a higher risk of asthma. In addition children of the teen’s age were associated with increased risks in the low SES group. For the crude proportions, factors such as (parental) smoking or having had no “contact with farm animals” were identified as strong risk factors for rhinitis.

**Significance:**

The “subscreen” package enabled the detection of notable subgroups for further investigations exemplarily for similar epidemiological research questions.

## Introduction

Since the consistency of study results across all participants cannot generally be assumed, analyses considering subgroups of participants are frequently used and discussed, with remarkably varying approaches [1–4]. However, conclusions about the true relationship between factors and a study endpoint can remain incomplete if not analyzed comprehensively. With increasing computer power and statistical state-of-the-art methodology, even a huge list of subgrouping factors can be investigated in an explorative manner. Various options for subgroup analyses, including graphical approaches, were presented in a systematic review of Ballarini et al. [5]. Among these, the subgroup screening with the R package “subscreen” [6] has demonstrated high efficiency and usefulness in the exploration of otherwise overlooked factors in clinical trials [7]. Any kind of statistical analysis, which was for instance pre-specified in an analysis plan such as difference in means or time-to-event analyses, can be done with the “subscreen” package for all subgroups simultaneously in one go and comprehensively visualized for further investigations. In contrast to e.g., statistical models with interaction terms, the basic approach of “subscreen” is to repeat the primary analysis for each subgroup. With the novel concept, we recommend fostering discussions in interdisciplinary teams (e.g., epidemiologists, clinicians, and statisticians) focusing on striking subgroups and evaluate interactively the relevance from a medical and statistical point of view.

From 2003 to 2006, a nationally representative survey was conducted on children and adolescents (later on often “children” for easier reading). This “German health interview and examination survey for children and adolescents (KiGGS)” aimed to improve the information available on the health of the up and -coming generation in Germany [8]. The subsequent KiGGS Wave 2 aimed to conduct a follow-up survey and had a data release in 2019 [9]. A huge amount of demographic, health-related, laboratory and environmental parameters (called “factors” in the following) including the socioeconomic status (SES) were collected [10]. Many potential risk and protective factors have already been investigated in various studies worldwide including socioeconomic variables [11, 12] indicating a higher risk for asthma and rhinitis in the case of a higher SES [13]. On the other hand, the results of KiGGS Wave 2 reported by Kuntz et al. [14] demonstrated that children with a low SES have a poorer level of general health and were more affected by asthma compared to their peers with a higher SES.

Even though the SES impact was less profound in the 12-month prevalence of bronchial asthma and allergic rhinitis compared to general health, we aimed to assess the scientific question of whether the effect of SES on asthma, rhinitis, or both is consistent among children when considering subgrouping factors. Additionally, the impact of these subgrouping factors on the crude proportions of these allergic diseases was investigated. For these aims, the R package “subscreen” as a graphical and analytical tool was used in this large epidemiological data set.

## Methods

### R package “subscreen” to investigate SES (in-)consistency

The second version of the “subscreen” package was illustrated with examples of clinical trials in Muysers et al. [7]. Several enhancements and additions were implemented with version 3 [6]. Basically, the “subscreen” package requires the specification of a user-defined statistical model which can be applied to one group, e.g., for time-to-event or proportion analyses, or to two groups, e.g., for the difference in means. In our case, the odds ratio (OR) describing the association between the disease (outcome) and the two SES groups (“low” or “high”) was calculated using logistic regression. The disease outcome (present or not) was asthma, rhinitis, or both. For each of the three outcomes and within each factor level combination (subgroup) the ORs were individually estimated.

This would be possible for all combinations of factor levels but for interpretational reasons, it was restricted to combinations of up to two-factor levels only. For example, 2 sex levels combined with 4 age groups resulted in 8 subgroups. Subsequent SES comparisons in each of these 8 groups in addition to the 6 single factor levels of gender (2) and age group (4), resulted in 14 ORs derived for the investigation and repeatedly used for each of the three disease outcomes. Empty cells are the result of a constellation of factor levels without children and consequently no OR were calculated.

For our presented purpose, a “striking factor” was defined on subjective assessments supported by various indicators. In line with the exploratory approach, we intentionally excluded the calculation of *p*-values and confidence intervals to avoid strict categorizations such as conventional significant and non-significant factors, instead of leaving room for interdisciplinary discussions of clinical relevance.

### R package “subscreen” to investigate crude proportions

To broaden the insight into the relationship of the investigated factors and the allergic diseases, crude proportions of all disease outcomes ignoring the SES status were additionally derived using the “subscreen” package. One element of the “subscreen” package is the variable importance (VIMP), which is based on the machine learning algorithm random forest [15–17]. VIMP was implemented to compensate for the intentional lack of confirmatory testing of subgrouping factors in “subscreen” [6]. While the VIMP feature within the “subscreen” package offers a ranked factor list of the importance only, to offer better insight, a more comprehensive analysis and result reporting outside the “subscreen” package was conducted in R. The random forest runs a series of decision trees, collect them and average their characteristics. An additional feature of the Random Forest is that it can collect the out-of-bag (OOB) sample [18]: at each tree iteration, the random forest leaves out observations and uses them to perform cross-validation and improve accuracy in the forest. VIMP was used to determine the factor (predictor) contribution in predicting the disease outcome (dependent variable) of interest. Minimal depth [19] was also used to compare the VIMP findings. Minimal Depth identifies the high impact predicting variables as the ones which partition the largest samples of the population. For the analysis, training and testing datasets were created, respectively using 80% and 20% of the observations.

### Exemplary further investigation of striking factors

While the explorations for both the SES-related and the crude proportions-related screening supported the finding of striking factors, a conclusion about their causal impact is not possible because of the post-hoc type of analyses and multiplicity reasons [20, 21]. Consequently, it is recommended to conduct further investigations, e.g., after the setting of a hypothesis, focusing on such striking factors. Since the term “striking” is in contrast to a “significance” approach with no strict classification, different hypotheses, and factor selections are obvious. For the purpose of this paper, a multivariable logistic regression analysis was derived exemplarily for a certain subjective selection of factors including interaction terms with SES to derive ORs and 95% CIs. As sensitivity analysis stepwise forward, backward, and bidirectional selection were conducted using the Akaike’s information criterion (AIC) as described in Harrell [22].

### Software references

SAS® version 9.4 and R [https://cran.r-project.org/] version 3.6.1 were used for the data set-up and supportive analyses. The R package “subscreen” version 3.0 was used for the subgroup screening [6]. The random forest was implemented by using the “randomForestSRC” R package [23].

### Study data

The KiGGS wave two studies comprised a nationwide, representative cross-sectional sample of children the age of 0–17 years from the Robert Koch Institute (RKI) [24]. A comprehensive overview of data architecture, collection, and management has been published [25]. Of the 15,023 children in the KiGGS Wave 2 data set, 4686 children were available for the analysis. Children aged 0–3 years (here an allergic disease diagnosis is mainly not reliable), children having a medium SES, children with missing information for asthma, rhinitis, or SES were excluded from the analysis dataset.

Missing values were handled as follows: (i) if asthma or rhinitis disease status or the SES category was missing, the child was withdrawn from the analyses, (ii) if a subgroup factor level (or the combination of two levels) did not have observations, it was set to the new category “no data” in order to avoid too many empty subgroups and to become aware of potential systematic missingness.

In the KiGGS survey, weighting variables were calculated to compensate for imbalances in certain variables with respect to the general German population of children and adolescents [25]. Since it is not the aim of the present analysis to estimate a population prevalence and because the sampling design is assumed to be non-informative, i.e., survey selection probabilities are not correlated with the outcome of factors [26], the weights were not used in our analyses.

### Outcomes

Three outcomes were investigated: (i) current asthma symptoms only (i.e., in last 12 months), (ii) current allergic rhinitis symptoms only (in last 12 months), (iii) both current asthma and current rhinitis (in last 12 months). This approach aimed to minimize inferences with overlapping associations for the three groups. The common control group included children who had neither current asthma nor current rhinitis. A “current” disease refers to the time frame “within the last 12 months” or “12-month prevalence” based on the KiGGS survey terminology [24]. The term “only” indicates the absence of the alternate disease. For improved readability throughout this article, “asthma” describes “current asthma only” and analogously for “rhinitis”. For each outcome the dichotomized status “yes” or “no” was taken from the source data of the RKI [24].

### SES

The assessment of the SES is based on a broad assessment of the parents’ education, occupational status, and income [10, 27]. From this, participants were categorized into three groups: “low”, “medium”, or “high” SES. In order to concentrate on presumed larger effects and for better usability of the ‘subscreen’ package, we compared children with low vs. high SES only in all analyses.

### Factors and subgroups

The selection of potentially relevant factors was not only based on the literature but also on any suspected association of the disease with SES and of their availability in the KiGGS dataset. In total, 87 factors were selected, including information on general health, quality of life [28], allergies, smoking, socio-demography and earnings, and breastfeeding. For simplicity, some factors were combined into new factors. Specifically, continuous factors or factors with numerous levels were categorized into few classes since the “subscreen” approach requires an ordinal or a nominal scale [7]. As a result, 56 factors comprising 221-factor levels were available. For a comprehensive overview, Supplementary Table S1 shows all used and modified factors. A “subgroup” is used to denote one specific level of a factor (e.g., “girls”) or one combination of two-factor levels (e.g., “girls of the youngest age group”).

## Results

Among the 4686 children, 78 (1.7 %) reported asthma only, 361 (7.7 %) rhinitis only, 72 (1.5 %) both asthma and rhinitis and 4175 (89.1%) neither disease. Crude ORs between outcomes and SES are presented in Table 1.

**Table 1:** Frequencies and percentages of disease outcomes (asthma and/or rhinitis) with crude odds ratios (OR) and Wald’s 95% confidence intervals (CI) for each socioeconomic status (SES) level.

	Asthma (*n* = 78)	Rhinitis (*n* = 361)	Asthma & Rhinitis (*n* = 72)	Control Group (*n* = 4175)	Total (*n* = 4686)
SES low *n* (%)	35 (44.9 %)	104 (28.8%)	24 (33.3%)	1361 (32.6%)	1524 (32.5%)
SES low *n* (%)	43 (55.1 %)	257 (71.2%)	48 (66.7%)	2814 (67.4%)	3162 (67.5%)
OR (95% CI)	1.68 (1.07–2.64)	0.84 (0.66–1.06)	1.03 (0.63–1.70)	–	

### Subgroup screening regarding SES

**Fig. 1:**
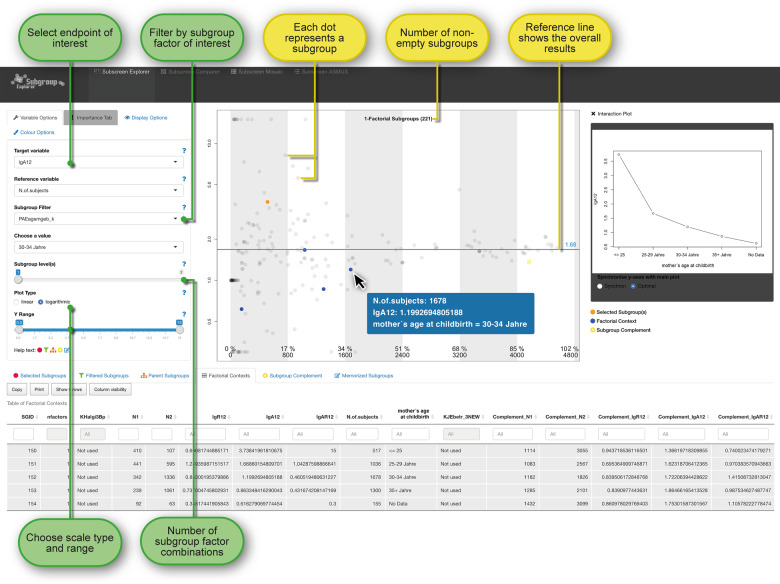
Each subgroup is represented by a dot in the central plot where the position is determined by the subgroup’s sample size (horizontal axis) and statistical measure of the treatment effect (vertical axis). The blue dots in the plot and numerical tabulated results below the plot for the factor “mother’s age at childbirth” appear after clicking the “Factorial Context” tab for a previously actively selected subgroup (orange). While the factorial context with blue dots highlights the individual levels of the factor “mother’s age at childbirth”, the yellow complementary group highlights the subgroup of all combined subjects excluding the subjects belonging to the selected (orange) subgroup. The interaction plot (line plot) at the right can be extended with another factor if desired.

**Fig. 2:**
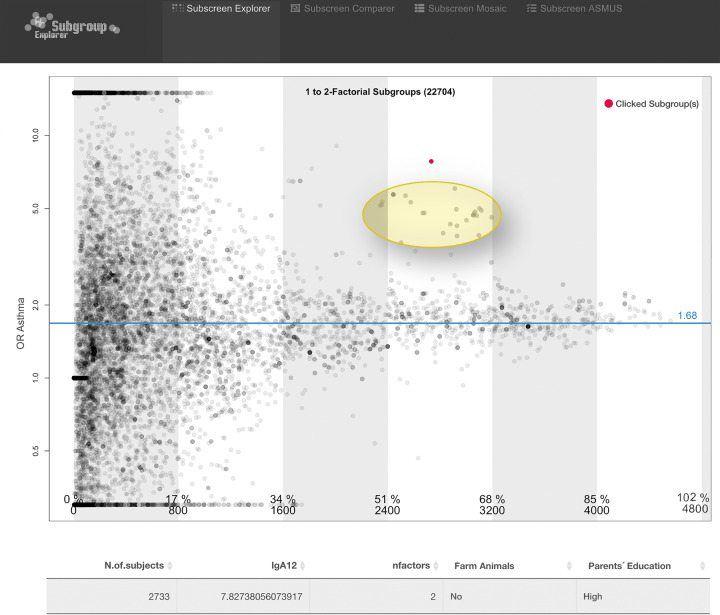
The 1–2 factorial subgroups for outcome asthma. The degree of dot transparency reflects the number of factor levels: the brighter the dot, the more factors are combined in that group. Darker dots can be also caused by the overlap of multiple subgroups. A visual striking subgroup is actively highlighted (red dot). A dot cluster (figure edited with a yellow oval) appears below the red highlighted subgroup. The striking cluster yellow oval added appears because other factor level combinations’ odds ratio, which includes parent’s education, are highly correlated with the red highlighted subgroup; while the combination with no “current contact with farm animals” yielded the highest odds ratio in that area.

For the subgroup screening, 221 1-factorial subgroups and 22,704 1–2 factorial subgroups (combinations of the 221-factor levels) were considered based on the 56 selected factors. Fig. 1 shows the surface of the “subscreen” package for the 1-level subgroups, with a first striking result of the factor “mother’s age at childbirth”. More than one million 1–3 factorial subgroups could be technically handled; however, already the 1–2 factorial subgroups in Fig. 2 offered a good display of the subgroup dispersion while 1–3 factorial subgroups would result in too much overlap of dots. On the other hand, striking subgroups consisting of a combination of three or even more factor levels are rather difficult to interpret and based often on very small group sample sizes; three or more factorial subgroups were thus omitted.

Throughout this subgroup screening, ORs greater than 1 indicates a stronger association of the children with low SES with the disease outcome in the respective subgroup. It happened that no observations occurred for a specific disease outcome and SES within a children subgroup. Such constellations but also subgroups with small numbers triggered extreme OR values. Such ORs were calculated but were truncated in the visualization in the “subscreen” tool at values 0.3 and 15 (individual user-specification) for an optimized view for all outcomes outside this range.

The new “interaction plot” feature in version 3.0 of “subscreen” [6] was helpful in identifying the factor “mother’s age at childbirth” as striking. The corresponding line plot at the top right in Fig. 1 shows a clear decreasing trend for the asthma outcome (n.b. the “No Data” group should not be considered for the trend). As age increases, the ORs decrease, reflecting a higher risk for the children of young women with a low SES to have asthma. In Fig. 1, the table below the dot plot shows simultaneously the numerical results and trend for the disease group both asthma and rhinitis (lgAR12). In addition, the rhinitis results (lgR12) are shown, though an OR trend is not available.

The line plots for the other disease outcomes and factors can be generated in a user-friendly way (not shown). For instance, we noticed high ORs for the low SES group for the asthma outcome for 11–13 years old children group (OR = 6.58) and for rhinitis in the 14–17 years old children group (OR = 1.13, whereas all other age groups have a consistent similar OR equal or below 0.62 for rhinitis).

Fig. 2 shows the extension of the subgroup screening to 1–2 level factorial subgroups, represented by 22,704 dots (cropped of sidebars for simplicity). A funnel shape is formed by the dispersion of the subgroup dots around the blue line representing overall asthma OR. Low sample size groups with high variation are shown on the left, whilst high sample size groups with low variation are shown on the right side of the graph. Deviations from this funnel shape indicate striking subgroups.

In Fig. 2, the red dot represents the high “Education level of parents” combined with no “current contact with farm animals”. An OR of 7.8 indicating a remarkable higher risk for asthma in children with a low SES is observed.

Other relevant factors (data not shown) interfering with the relationship of SES and all three disease outcomes were the allergy factors SX1-screening, air/pollen, and food allergy. These factors showed high ORs in either direction for different levels but lack a clear trend-making hypothesis generation difficult. In addition, the subgroup “Berlin” residency showed a lower risk for the low SES group for all disease outcomes; though, this subgroup had only a few observations.

Overall, the subgroup screening showed larger OR dispersion for the subgroups for asthma compared to rhinitis indicating a general stronger impact of the various factors on the relationship between asthma and SES than on rhinitis and SES. This can easily be seen with the “Compare” feature of the “subscreen” package showing a “Bubble Plot” (Supplementary Fig. S1). The OR dispersion of subgroups of “both asthma and rhinitis” is in-between asthma’s and the rhinitis OR dispersion (data not shown).

### Subgroup screening investigating crude disease proportions

**Fig. 3:**
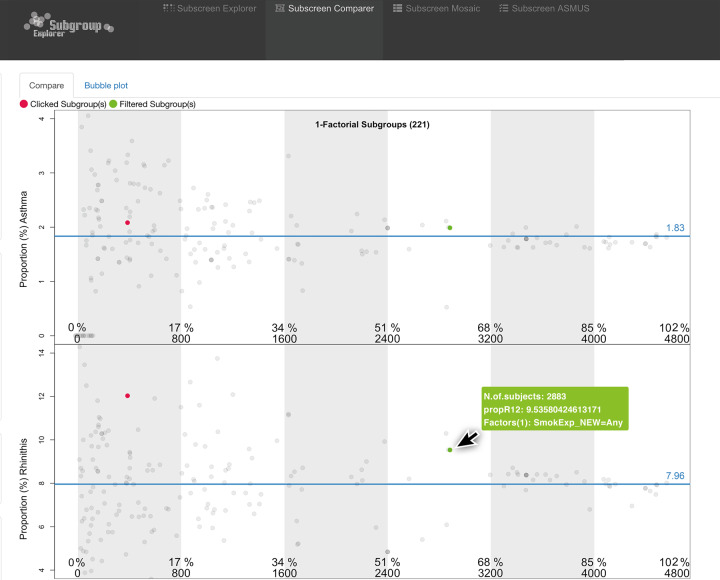
The proportions of children with asthma (upper panel) and rhinitis (lower panel) are simultaneously shown for all 221-factor levels and compared to the overall proportions (blue lines). Factors “Any smoke exposure” (green dots) and “Active smoke exposure” (red dots) are selected.

Crude proportions, independent of the SES interaction and the OR plotting, were simultaneously visualized for the subgroups for asthma and for rhinitis using the “Compare” feature. As seen in Fig. 3 the “yes”-level of the factor “Any smoke exposure” (green dots) shows a remarkably higher proportion for rhinitis (9.5%) but only a moderately increased proportion for asthma compared to their averages of 8.0% and 1.8%, respectively. In a smaller subset, “Active smoke exposure” (red dots) showed for rhinitis an extremely higher proportion (12%) if the corresponding youths were smoking. Similar results were observed for the factor “Smoking during breastfeeding” as reported by their mothers (data not shown).

Furthermore, the “no”-level for the factor “contact with farm animals in the age of 0–6 years” resulted in a remarkably high proportion of 10.3% of children having rhinitis (not highlighted in the figure).

### Random forest

Some variables were highly correlated and were thus excluded from the analyses (“correloplot” in Supplementary Fig. S2). For each disease outcome, a separate Random Forest model was run. Missing observations were omitted and not imputed because a lower OBB error rate estimate and a higher variance explained were observed in a sensitivity analysis.

**Fig. 4:**
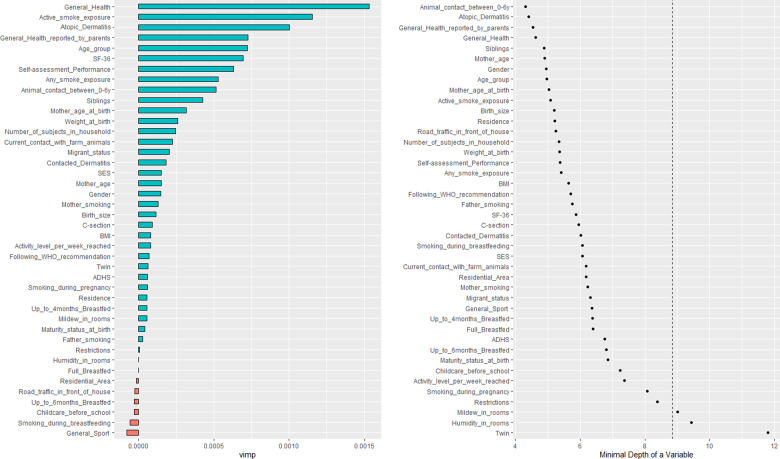
Variable Importance (VIMP) for the best-fitted random forest (left) is presented for rhinitis. Blue bars indicate the importance of variables (positive VIMP) relative to their lengths, red bars indicate presumably noise variables (negative VIMP). The best combination for the hyper-parameters was found to be nodesize = 10, nsplit = 2, and mtry = 3 with the lowest OBB error estimate of 2%. Minimal depth (right) refers to the topology of the forest. Low minimal depth indicates important variables. The dashed line represents the threshold of the maximum value for variable selection.

The most important variables regarding VIMP identified by the Random Forest associated with rhinitis were “General Health”, “Active smoke exposure”, “Atopic Dermatitis” and “Age group” (Fig. 4, left). However, the Random Forest does not give an indication of the direction of the effect. Minimal depth showed for rhinitis similar results to VIMP, whereas the highest impact variables to partition the data were found to be “Animal contact between 0 and 6 years” and “Atopic Dermatitis” (Fig. 4, right). The corresponding results for asthma and both asthma and rhinitis are shown in Supplementary Figs. S3 and S4, respectively.

### Exemplary further investigation of striking factors

A multivariable logistic regression model was run for the factors “Mother’s age at birth”, “Age group”, “Current contact with farm animals”, “Smoking during breastfeeding”, and “Residence”, including their interaction with SES. Numerically similar results were found for (i) using the forward selection and the full model as well as (ii) using the backward elimination and the bi-directional approach. We thus report only the forward selection and the backward elimination models (Supplementary Table S2) representing all four approaches. The reported *p*-values for each outcome describe the given model test compared with the intercept-only model.

Asthma (*p* = 0.106): ORs with a nominal *p*-value below 5% were observed only for the SES interactions of age groups with an OR of 0.20 (0.04–0.98; 95% CI) for “14–17 years” and 0.19 (0.04–0.88) for “7–10 years” where the “11–13 years” group was used as the reference group.

Rhinitis (*p* < 0.001): one OR with a nominal *p*-value below 5% was 2.22 (1.23–4.01) observed for “Mother’s age at birth” for the age group “up to 24 years” with “25–29 years” as reference.

Both asthma and rhinitis (*p* < 0.001): no single OR with a nominal *p*-value below 5% was seen.

## Discussion

### Main findings

Our results based on the crude OR indicated a considerably increased asthma risk for children with a low SES, whereas, for rhinitis, we found an increased risk for children with a high SES. No such risk trend was observed for children with both asthma and rhinitis, most probably since the odds were outbalanced by the diverging trends of asthma and rhinitis.

The subgroup screening was applied to find amplifying or weakening factors that might interfere with the association of disease (asthma, rhinitis, or both) with SES. Asthma demonstrated higher variation across all subgrouping factors and more striking factors were identified compared to rhinitis. One finding triggered the hypothesis of a higher risk of children’s asthma for less privileged mothers at a younger age. This finding was based on both (i) observed subgroups at the edge of the funnel-shaped plot and (ii) the monotonicity trend in the interaction plot. The combined disease outcome, i.e., both current asthma and rhinitis, showed huge variations (picture not shown), and no clear striking factors were identified.

Furthermore, remarkably increased ORs were observed for the low SES group: (i) for the asthma outcome for 11–13-year old children (OR = 6.58) and (ii) for rhinitis outcome for the 14–17-year old children group (OR = 1.13, whereas all other age groups have an OR equal or below 0.62). A lack of compliance, in terms of appropriate disease management, could be hypothesized for the youths of the low SES group and requires further investigations.

High “Education level of parents” in combination with no “current contact with farm animals” was found to increase the risk of asthma for the low SES group. However, considering the two factors, an interaction is questionable from a content point of view and requires further investigation; furthermore, the factor level of high education as opposed to the low SES group.

The crude proportions were simultaneously inspected with the subgroup screening for asthma and for rhinitis. Smoking in different variants (any or active exposure and smoking during breastfeeding) seems to increase the proportion of children experiencing rhinitis. Similarly, when no “contact with farm animals in the age of 0–6 years” was experienced, a remarkably higher proportion of children having rhinitis was observed. Random forests presented graphically which factors contribute more in predicting the dependent variables. Though not showing the direction of the most important variable found, the random forest was able to confirm striking factors from the subgroup screening, e.g., for smoking and animal contact-related factors. Furthermore, other factors were derived as important for the prediction of rhinitis, e.g., “General health” or “Atopic Dermatitis”. Noteworthy, all three random forest models had a poor predictive ability and showed low variance explained.

Interesting factors from the explorative investigations were used for a statistical adjusted logistic regression model including interaction terms with the SES. For asthma, the forward selection logistic model yielded considerable ORs for the SES interactions with age groups in line with observations for a higher risk for children in the age group 11–13 years and the low SES. A meaningful OR was observed for “Mother’s age at birth” in the youngest age group for rhinitis with the forward selection model as already seen in the subgroup screening. Therefore, the logistic regression model partially confirmed what was found in the subgroup screening.

### Strengths and limitations

The “subscreen” package had already demonstrated its usefulness and efficiency to detect potentially overlooked factors in clinical trials [7]. It was conveniently extended here to a large population-based epidemiologic study given the tool’s property to handle huge data sets with millions of subgroups. The usually lengthy and tedious explorative search for striking subgroups was replaced by an interactive, comprehensive, and coherent screening.

The combination of disease outcomes both asthma and rhinitis seemed to increase the dispersion of the investigational outcomes (ORs) but striking factors leading to a hypothesis generation were not detected. This might be due to the outbalancing of the observed diverging effect of risk trends in asthma and rhinitis. While this composite group appears not informative, the disjunction of the disease groups asthma and rhinitis were beneficial to avoid overlapping vanishing effects for the investigations in these two disease outcome groups.

In order to report a prevalence and to make conclusions about the whole target, population weighting is generally recommended. Since the sampling design for the KiGGS survey is assumed to be not informative, the weights were not essential for our analyses [26] and therefore omitted. Furthermore, we did not aim to describe the prevalence in German childhood but to increase the understanding of the data and allow hypothesis generation.

To find further explaining factors for the association between allergy outcomes and SES, a discriminating approach was used. For this, only the low and high SES groups were considered while children of the medium SES were dismissed. A U-shaped distribution, i.e., the extreme outcome of the medium SES group, deemed not existing based on the monotonicity of ORs for asthma in conjunction with SES [14]. Monotonicity of ORs for rhinitis was not observed [14], however, the focus of our investigation was on the screening for additional and interacting factors independent of the OR’s monotonicity for the SES groups. Alternatives to dismissing the medium group, such as dichotomization, would imply less comparability with other publications.

Due to missing data and/or few occurrences of diseases within a factor level, e.g., for the factor “Mildew in rooms”, the number of outcome events was very small. These factors were nevertheless kept in the screening in order to detect extreme abnormalities. However, low associations seen in this data do not necessarily mean that no association exists.

## Conclusions

The easy-to-use “subscreen” tool can handle large epidemiologic study data with millions of subgroups fast and efficiently. This has been enhanced in a new version of the tool. It now includes further supportive graphical and analytical features such as interaction plots facilitating also trend analyses. With the novel concept, we propose to make use of this fast and efficient tool in an interdisciplinary team. The common discussion from different fields of expertise of striking subgroups can lead directly to the generation of new hypotheses or confirmation of previous assumptions. This exploratory approach yields more numerical and graphical insight into the data compared to the restricted use of a few selected subgroups.

For rhinitis, our approach confirmed findings for known risk factors (such as smoking) and protective factors (such as contact with farm animals). For rhinitis and for asthma, unexpected findings were seen for factors in conjunction with SES such as the age of both children and mothers. Specifically for asthma, our findings could trigger the hypothesis of a higher risk for children from less privileged, younger mothers. However, further investigations are needed to support this hypothesis.

In summary, the “subscreen” tool be can be applied routinely to clinical and epidemiological study data for future discussions and hypothesis generation requiring further analyses.

## Supplementary information

Supplementary material

## Data Availability

Used source data, i.e., The German Health Survey for Children and Adolescents (KiGGS Wave 2), were provided by the Robert Koch Institute, Department of Epidemiology and Health Monitoring [24]; data are available from the Robert Koch Institute.

## Code availability

The software SAS® Version 9.4 and R Version 3.6.1 with the free of charge “subscreen” package version 3.0 [6] was used. Programming code is available for the Journal upon request by CM.
