# Supplementary material for: A novel concept of screening for subgrouping factors for the association between socioeconomic status and respiratory allergies
Source: J Expo Sci Environ Epidemiol. 2021 Jul 12;32(2):295–302. doi: 10.1038/s41370-021-00365-x (PMC8920883; doi:10.1038/s41370-021-00365-x)
Supplement: Supplementary file 1 — Supplementary material [file 41370_2021_365_MOESM1_ESM.pdf]

Supplementary material

A Novel Concept of Screening for Subgrouping Factors for the Association Between Socioeconomic Status and Respiratory Allergies

Supplementary Figures

Supplementary Figure S1

Overview of the 221 1-factorial subgroups as Bubble Plot where the size of the dots displays the subgroup size. The coordinates of the dot represents the SES Odds Ratio for asthma on the horizontal axis and the SES Odds Ratio for rhinitis on the vertical axis. The subgroup 'mother's age at childbirth' on the level youngest age group is discussed in Figure 1 of the main article as interesting factor (green dot) while the subgroup highlighted as red dot shows a remarkable high OR for asthma which is the youths group 11-13 years. Noteworthy, the dispersion of the OR for asthma is stronger compared to the OR dispersion of rhinitis; with other words, more striking subgroups resulted in our screening for the disease outcome asthma than for rhinitis.

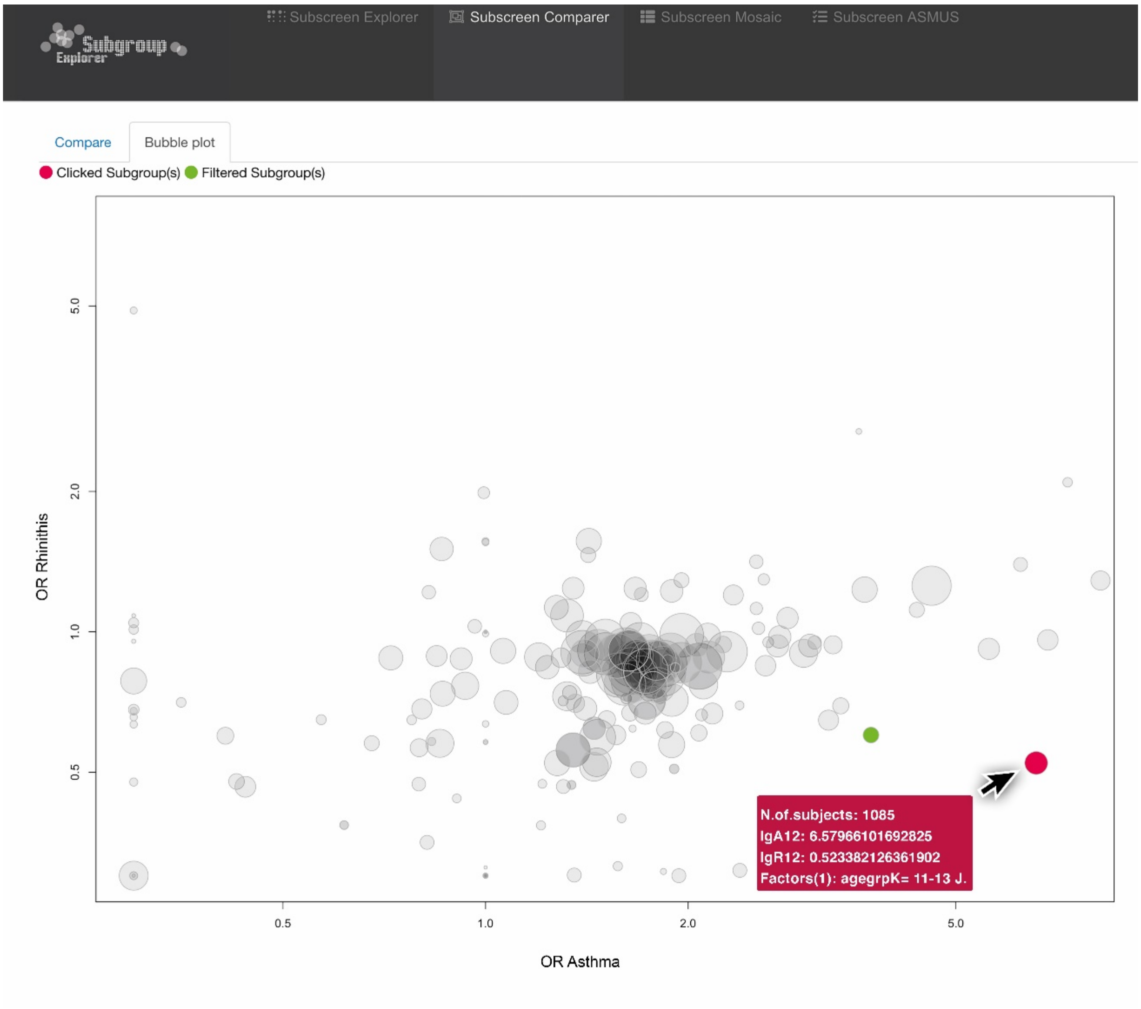

Supplementary Figure S2

Correlogram for the variables in the dataset. The graph shows the upper matrix of the results of the correlation between the used variables in the dataset. Red full circles indicate a negative correlation, whilst blue full circles indicate a positive correlation. The higher the transparency the lower the absolute correlation value. All the elements on the main diagonal were removed as the correlation value was 1. Some variables were highly correlated. For this reason and to avoid multicollinearity in our results of the Random Forest analyses, variables with a correlation higher than 0.65 in absolute value were excluded from the analysis dataset. As a result, six variables were excluded from the analysis, which were 'Restrictions due to disease', 'Air pollen allergy', 'Passive smoke exposure', 'Breastfed', 'Education level of parents' and 'Income categories'.

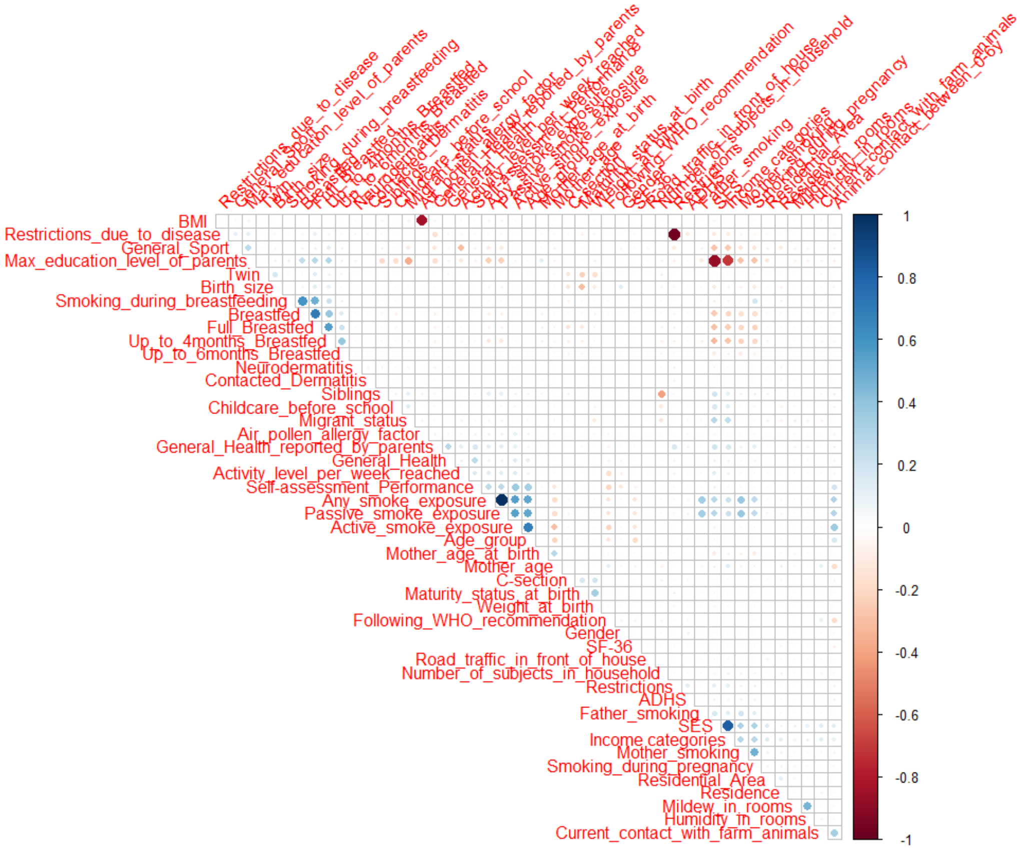

Information for both Supplementary Figure S3 and S4

Random Forests were implemented by using 'randomForestSRC' R package introduced by Ishwaran et al. (2008). To obtain the best model three hyper-parameters were tuned.

- *mtry* represents the number of variables available for splitting at each tree node
- *nsplit* specifies the number of random split points at which an x-variable is tested
- *nodeside* indicates the average node size over the forest.

The Out-of-bag sample (Breiman, 1996) was used to tune the parameters using different combinations of the hyper-parameters. A total of 2310 combinations were identified using nodesize (10, 20, 35, 50, 70, 85, 100, 120, 150, 180, 190, 200, 210, 220) , mtry (1, 2, 3, 4, 5, 6, 7, 8, 9, 10 ,15 , 20, 35, 40, 50) and nsplit equal to (2, 3, 4, 5, 6, 7, 8, 9, 10, 15, 20).

Supplementary Figure S3

For the disease outcome (dependent variable) 'asthma', the best combination for the hyper-parameters was found to be nodesize=10, nsplit= 2 and mtry=3, with the lowest OBB error estimate of 2%. VIMP and minimal depth use different criteria to select variables, therefore the ranking is expected to be somewhat different. In this case, both VIMP and Minimal depth give very similar results. It can be observed that the most important variables identified by the Random Forest to predict asthma are 'General Health reported by Parents', 'ADHS' and 'number of subjects in the household'. Minimal depth considers the same variables identified by the VIMP as the most powerful in partitioning the dataset.

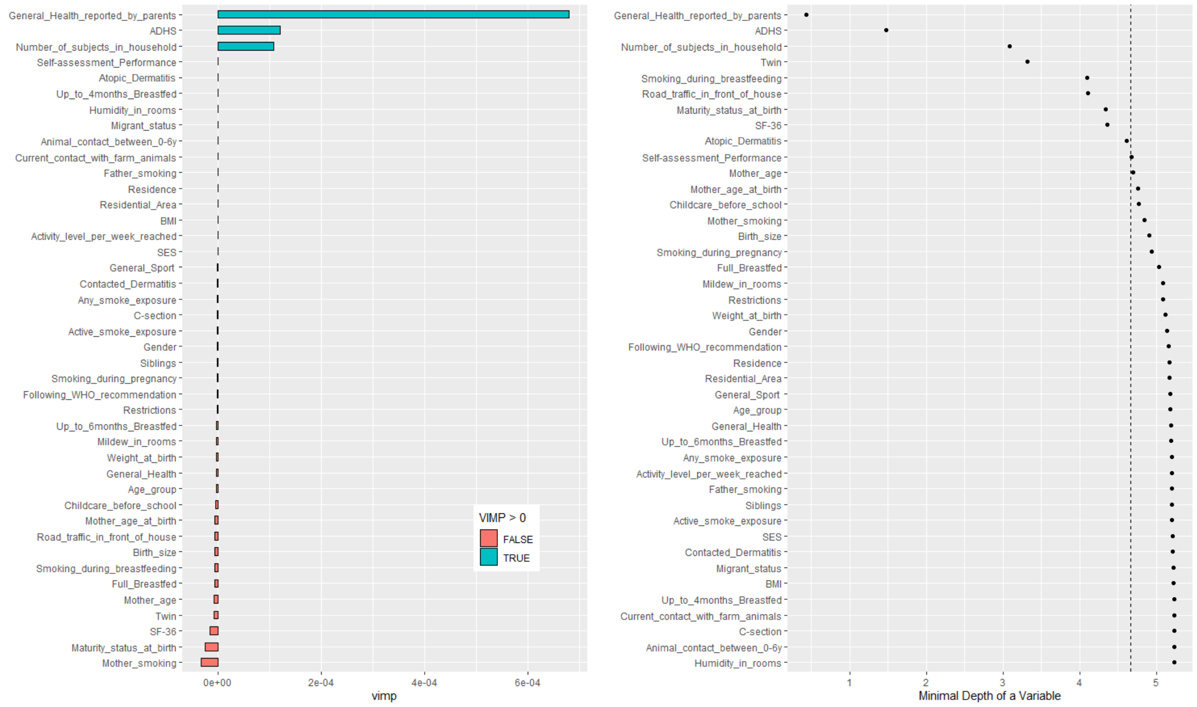

Supplementary Figure S4

For the disease outcome (dependent variable) ‘rhinitis and asthma’, the best combination for the hyper-parameters was found to be nodesize=10, nsplit= 3 and mtry=6, with the lowest OBB error estimate of 2%. It can be observed that the most important variables identified by the RF to predict having both rhinitis and asthma are General Health, Active smoke exposure, Atopic Dermatitis. Minimal depth, on the other hand, considers Animal contact between 0-6 years and Atopic Dermatitis as the most powerful variables to partition the dataset.

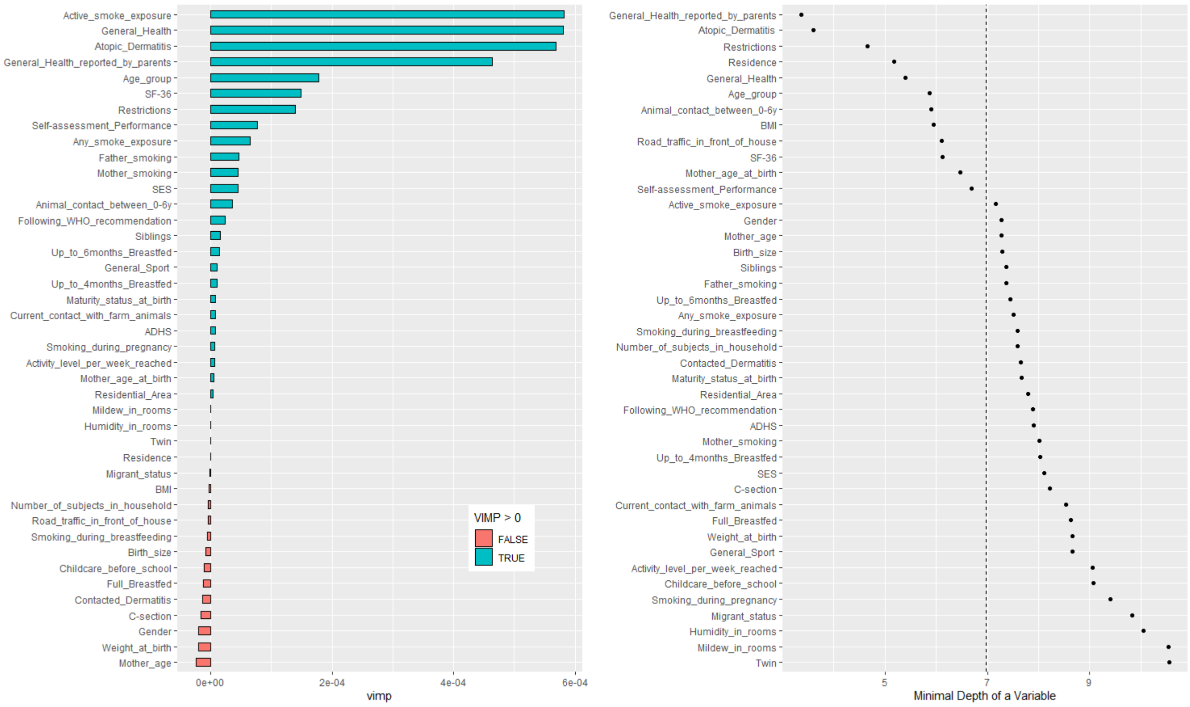

Supplementary Tables

Supplementary Table S1

All original* and transformed factors used at a glance. The column 'Original factor name' shows the KiGGS Wave 2 database terminology of the variables. Since the database and its explanations are German, a brief translation is provided in the last column 'Used factor and its level'. If the variables were used in our analyses without modification the column 'Categorization / algorithm' states 'unchanged', otherwise the transformation rule is provided.

Category	Original* category, label and ref number	Original* factor name	Categorization / algorithm, if applicable**	Used factor and its levels
General Health	Gesundheitszustand allgemein (110200)	a) GZEmehm1_k2<br>b) GZmehm1_k2<br>c) GZcshcn3<br>d) GZ3cshcn3a	unchanged	a) General health reported by parents<br>b) General health<br>*each on three ordinal levels*<br>c) Restrictions<br>d) Restrictions due to disease<br>*each with 'yes, no' level*
Allergy	Allergien (110190)	a) KHalgi3Bp<br>b) KHalgi2p	unchanged	a) atopic dermatitis<br>b) contact dermatitis<br>*each with 'yes, no' level*
Quality of Life***	Lebensqualität allgemein (110900)	a) LQEkidsB<br>b) LQsf361	a) divide into three levels<br>b) unchanged	a) Kidscreen-10 Index (EFB)<br>b) SF-36<br>*each with three ordinal levels*
Allergy	Allergologie (120201)	a) LBalge1_s, LBalge5_s, LBalgg12_s, LBalgg213_s, LBalgg214_s, LBalgg6_s, LBalgm2_s, LBalgt215_s, LBalgt3_s, LBalgw230_s, LBalgw231_s, LBalgw6_s, LBalgsx1_s<br>b) LBalgf1_s, LBalgf233_s, LBalgf431_s, LBalgf432_s, LBalgf13_s, LBalgf14_s, LBalgf17_s, LBalgf439_s, LBalgf440_s, LBalgf423_s<br>c) LBalgsx1_s	a) and b)<br>• Yes, if any factor is yes<br>• No, if all factors are no<br>• else missing (e.g. 'not available' or 'missing')<br><br>c) unchanged	a) Air/pollen allergy factor<br>b) Food allergy factor<br>c) SX1-Screening<br>*each with 'yes, no' level*
ADHS	ADHS (130104)	PKEadhs	unchanged	ADHS diagnosis<br>*with 'yes, no' level*
Physical activity	Körperliche Aktivität allgemein (150100)	a) KAleist<br>b) KAempf_who<br>c) KAempf_inakt<br>d) KAspoB	unchanged	a) Performance, self-assessment *with ordinal levels*<br>b) Following WHO recommendation *(yes, no)*<br>c) Activity level per week reached *(yes, no)*<br>d) General sport *(yes, no)*
Smoking	Rauchen allgemein (150500)	a) RCErauch2mX<br>b) RCErauch2vX<br>c) RCEschwX<br>d) RCEstillX<br>e) RCtaeglX, RCstatBX, RCshi12X, RCstatCX, RCstarkX<br>f) RCErauchmX, RCErauchvX, RCErauch2mX, RCEpass3X, RCEschwX, RCEstillX, RCfreundX, RCpass1BX, RCortX<br>g) a)-g) for 'any exposure' evaluation	All factors were, if not already, condensed to the outcomes 'any, no, no info'<br><br>Three new factors were defined. First factor has outcome 'any' if any smoking variable has an 'any' outcome, 'no' if all outcomes were 'no', and 'no info' otherwise. The second factor analogue for active exposure (RCtaeglX, RCstatBX, RCshi12X, RCstatCX, RCstarkX). The third factor analogue for passive exposure (RCErauchmX, RCErauchvX, RCErauch2mX, RCEpass3X, RCEschwX, RCEstillX, RCfreundX, RCpass1BX, RCortX)<br>The factors RCErauch2mX, RCErauch2vX, RCEschwX, RCEstillX were kept as single factors in addition	a) Mother smoking<br>b) Father smoking<br>c) Smoking during pregnancy<br>d) Smoking during breastfeeding<br>e) Active smoke exposure<br>f) Passive smoke exposure<br>g) Any smoke exposure<br><br>*each with nominal levels 'any, no, no info'*

Category	Original* category, label and ref number	Original* factor name	Categorization / algorithm, if applicable**	Used factor and its levels
Habitation and Environment	Wohnen und Umwelt (160400)	a) WUEtier7<br>b) WUtier8<br>c) WUschimmB<br>d) WUfeucht<br>e) WUsubj<br>f) WUstrasse	unchanged	a) current contact with farm animals<br>b) contact with farm animals in the age of 0-6 yrs<br>c) Mildew in rooms<br>d) Humidity in rooms<br>*each „yes, no'*<br>e) residential area *with four nominal levels*<br>f) Road traffic in front of house/building *with six nominal levels*
Age and Gender	Alter und Geschlecht (170100)	a) agegrpK, age2<br>b) sexa	dismissing children in the age of 0-3 yrs, otherwise unchanged	a) Age *with four ordinal levels*<br>b) Gender *with two levels*
Perinatal Factors / Pregnancy and Birth	Perinatale Faktoren/Schwangerschaft und Geburt (170302)	a) KJEgebgroe_k<br>b) KJEgebreife<br>c) KJsect<br>d) KJEgebgewi_k	a) made up of previous seven levels<br>b) – d) unchanged	a) Birth size *with three ordinal levels*<br>b) Maturity status at birth *with nominal levels*<br>c) C-section *with nominal levels*<br>d) Weight at birth *with ordinal levels*
Migratory Status	Migrationsstatus (170500)	Mimigrant	unchanged	Migrant status *(yes, no , no info)*
Origin	Stamm (180100)	wob	unchanged	Residence *with nominal levels (West, East, Berlin)*
Childcare, School	Kinderbetreuung, Schule (170303)	a) KJEbetr_a and KJEbetr_mm<br>b) KJEbetr	a) cumulated year levels<br>b) unchanged	a) Age at start of childcare *with three ordinal year levels*<br>b) Childcare before school only within family *(yes, no)*
Socio-demography and earnings	Soziodemografie allgemein, Einkommen (170400)	a) SDEeinkB_k<br>b) SDEisced97eu	a) cumulated in classes '1-10', '11-12', '13'<br>b) unchanged	a) Income categories *with three ordinal year levels*<br>b) Education level of parents *with ordinal level groups*
Family status, number of children, and siblings	Familienstand, Kinderzahl, Geschwisterzahl (170201)	a) PAEgeschw1zB and PAEgeschw2zB<br>b) PAEzwi<br>c) PAEhh_k<br>d) PAEagemgeb_k<br>e) PAEagem_k<br>f) PAEagev_k	a) condensed<br>b) – f) unchanged	a) Number of siblings *(0, 1, >1)*<br>b) Twin *(yes, no)*<br>c) Number of subjects in household *with ordinal classes*<br>d) Mother's age at birth *with ordinal levels*<br>e) Mother's age *with ordinal levels*<br>f) Father's age *with ordinal levels*
Anthropometry	Anthropometrie (120110)	a) USgroe_st<br>b) USgewi_st<br>c) USbmi_iotf	divide into three levels	a) Height<br>b) Weight<br>c) BMI<br>*each with three ordinal levels*
General Breastfeeding	Stillen allgemein (150600)	a) SNEstill2_mm<br>b) SNEstillv_mm<br>c) SNEstill<br>d) SNEstillv<br>e) SNEstillv4<br>f) SNEstillv6	a) and b) divide into three levels<br>c) – f) unchanged	a) Breastfeeding (Bf) up to which child's age *with ordinal levels*<br>b) Full Bf up to which child's age *with ordinal levels*<br>c) Ever Bf *(yes, no)*<br>d) Full Bf *(yes, no)*<br>e) Up to 4th month full *Bf (yes, no)*<br>f) Up to 6th month full Bf *(yes, no)*

* - Robert Koch Institute (2019)
- Mauz (2017)

** 'divide into three': using the 1/3 and 2/3 quantile as cut-points based on outcomes actually used for analyses
'unchanged': using the outcome as delivered in the original* database

*** Further Quality of Life information are available in The KIDSCRREN (2020)

Supplementary Table S2

Excerpt of Odds ratios from logistic backward elimination model. ‘Missing Data’ groups are omitted because of sparse or even no outcome events. Analogously to the truncated subgroup ORs in the subscreen Figures 1 and Figures 2 of the main article, extreme ORs occurred due to no or sparse outcome event numbers.

Disease Outcome	Factor level [a]	Odds Ratio [b]	95% Confidence Interval [b]
Asthma (p = 0.009)	Intercept	67.31	(32.78 - 138.19)
	SES[a]	1.82	(1.16 - 2.87)
	crnt contact farm animals (No)	0.49	(0.24 - 1.04)
Rhinitis (p < 0.001)	Intercept	15.45	(8.59 - 27.80)
	SES[a]	0.76	(0.36 - 1.64)
	Age group (14-17 yrs)	0.48	(0.29 - 0.81)
	Age group (4-6 yrs)	4.09	(1.50 - 11.15)
	Age group (7-10 yrs)	1.66	(0.85 - 3.23)
	crnt contact farm animals (No)	0.89	(0.54 - 1.46)
	SES[a] :Age group (14-17 yrs)	2.19	(1.19 - 4.04)
	SES[a] :Age group (4-6 yrs)	1.09	(0.36 - 3.33)
	SES[a] :Age group (7-10 yrs)	0.98	(0.46 - 2.05)
	SES[a] :crnt contact farm animals (No)	0.66	(0.33 - 1.32)
Both Asthma and Rhinitis (p < 0.001)	Intercept	> 999	(0.00 - > 999)
	SES[a]	0.00	(0.00 - > 999)
	Mother’s age at birth (30-34 yrs)	2.58	(0.69 - 9.68)
	Mother’s age at birth (≥ 35 yrs)	5.37	(0.67 - 42.85)
	Mother’s age at birth (≤ 24 yrs)	0.92	(0.37 - 2.26)
	Age group (14-17 yrs)	0.84	(0.48 - 1.48)
	Age group (4-6 yrs)	4.71	(1.77 - 12.53)
	Age group (7-10 yrs)	2.00	(1.03 - 3.89)
	crnt contact farm animals (No)	0.36	(0.14 - 0.91)
	Residence (East)	0.00	(0.00 - > 999)
	Residence (West)	0.00	(0.00 - > 999)
	SES[a] :Mother’s age at birth (30-34 yrs)	0.40	(0.09 - 1.77)
	SES[a] :Mother’s age at birth (≥ 35 yrs)	0.34	(0.04 - 3.23)
	SES[a] :Mother’s age at birth (≤ 24 yrs)	> 999	(0.00 - > 999)
	SES[a] :Residence (East)	> 999	(0.00 - > 999)
	SES[a] :Residence (West)	> 999	(0.00 - > 999)

[a] The estimated group level is stated in parenthesis. The reference level is the only not mentioned level of that factor (cf. Supplementary Table S1). SES has always the low group level as reference. Thus, ORs greater than 1 indicate a stronger association of the children with low SES with the disease outcome. The symbol ‘:’ indicates the interaction term of two factors

[b] ORs and CI limits greater than 999 or labeled by R package *AICcmodavg* with infinity (‘Inf’) are harmonized in this table as ‘> 999’.

References in Supplementary Material section

- Breiman L. Out-of-bag estimation. Technical report, Dept. of Statistics, Univ. of Calif., Berkeley. 1996. https://www.stat.berkeley.edu/~breiman/OOBestimation.pdf

- Ishwaran H, Kogalur UB, Blackstone EH, Lauer MS. Random Survival Forests. The Annals of Applied Statistics. 2008; 841–860

- Mauz E, Gößwald A, Kamtsiuris P, Hoffmann R, Lange M, Schenck U et al.. New data for action. Data collection for KiGGS Wave 2 has been completed. Journal of Health Monitoring. 2017; 2(S3):2–27. DOI 10.17886/RKI-GBE-2017-105

- Robert Koch Institute. The German Health Survey for Children and Adolescents (KiGGS Wave 2). Department of Epidemiology and Health Monitoring. Public Use File first version. 2019. Accessed Oct 27 2019; doi: 10.7797/17-201417-1-1-1

- The KIDSCRREN Group Europe (Ed). The KIDSCREEN Questionnaires: Quality of life questionnaires for children and adolescents. Handbook. Pabst Science Publishers, Lengerich; 2020
